# Supplementary material for: Development of a Multiplex Quantitative PCR for Detecting Porcine Epidemic Diarrhea Virus, Transmissible Gastroenteritis Virus, and Porcine Deltacoronavirus Simultaneously in China
Source: Vet Sci. 2023 Jun 18;10(6):402. doi: 10.3390/vetsci10060402 (PMC10301166; doi:10.3390/vetsci10060402)
Supplement: Supplementary file 1 [file vetsci-10-00402-s001.zip › vetsci-2438862-supplementary.pdf]

**Table S1.** Results of PCR and qPCR amplification of 462 samples.

| Samples | Province | qPCR | | | PCR | | |
|---|---|---|---|---|---|---|---|
| | | PEDV | TGEV | PDCoV | PEDV | TGEV | PDCoV |
| 1 | Jiangsu | + | + | + | + | + | + |
| 2 | Jiangsu | + | + | + | + | + | + |
| 3 | Jiangsu | + | + | + | + | + | + |
| 4 | Jiangsu | + | + | + | + | + | + |
| 5 | Jiangsu | + | + | + | + | + | + |
| 6 | Jiangsu | + | + | + | + | + | + |
| 7 | Jiangsu | + | + | + | + | + | + |
| 8 | Jiangsu | + | + | + | - | + | + |
| 9 | Jiangsu | + | + | + | + | - | + |
| 10 | Jiangsu | + | + | + | + | + | + |
| 11 | Jiangsu | + | + | + | + | + | + |
| 12 | Jiangsu | + | + | + | + | + | + |
| 13 | Jiangsu | + | + | + | + | + | + |
| 14 | Jiangsu | + | + | + | - | + | + |
| 15 | Jiangsu | + | + | + | + | + | + |
| 16 | Jiangsu | + | + | + | + | + | + |
| 17 | Jiangsu | + | + | + | + | + | + |
| 18 | Jiangsu | + | + | + | + | + | + |
| 19 | Jiangsu | + | + | + | + | + | + |
| 20 | Jiangsu | + | + | + | + | - | + |
| 21 | Jiangsu | + | + | + | + | + | + |
| 22 | Jiangsu | + | + | + | + | - | + |
| 23 | Jiangsu | + | + | + | + | + | + |
| 24 | Jiangsu | + | + | + | + | + | + |
| 25 | Jiangsu | - | - | - | - | - | - |
| 26 | Jiangsu | - | - | - | - | - | - |
| 27 | Jiangsu | + | - | + | + | - | + |
| 28 | Jiangsu | + | - | + | + | - | + |
| 29 | Jiangsu | + | - | + | + | - | + |
| 30 | Jiangsu | - | - | - | - | - | - |
| 31 | Jiangsu | - | - | - | - | - | - |
| 32 | Jiangsu | - | - | + | - | - | + |
| 33 | Jiangsu | + | - | + | + | - | + |
| 34 | Jiangsu | - | - | + | - | - | + |
| 35 | Jiangsu | + | - | + | + | - | + |
| 36 | Jiangsu | + | - | + | + | - | + |
| 37 | Jiangsu | + | - | + | + | - | + |
| 38 | Jiangsu | + | - | + | + | - | + |
| 39 | Jiangsu | - | - | - | - | - | - |
| 40 | Jiangsu | + | - | + | - | - | + |
| 41 | Jiangsu | + | - | - | - | - | - |

| Samples | Province | qPCR | | | PCR | | |
|---|---|---|---|---|---|---|---|
| | | PEDV | TGEV | PDCoV | PEDV | TGEV | PDCoV |
| 42 | Jiangsu | - | - | - | - | - | - |
| 43 | Jiangsu | + | + | + | - | + | + |
| 44 | Jiangsu | - | - | - | - | - | - |
| 45 | Jiangsu | - | - | + | - | - | + |
| 46 | Jiangsu | + | - | + | - | - | + |
| 47 | Jiangsu | + | + | + | - | - | + |
| 48 | Jiangsu | + | - | + | - | - | - |
| 49 | Jiangsu | - | - | + | - | - | + |
| 50 | Jiangsu | - | - | + | - | - | + |
| 51 | Jiangsu | - | - | - | - | - | - |
| 52 | Jiangsu | - | - | - | - | - | - |
| 53 | Jiangsu | - | - | + | - | - | + |
| 54 | Jiangsu | - | - | - | - | - | - |
| 55 | Jiangsu | - | - | - | - | - | - |
| 56 | Jiangsu | - | - | - | - | - | - |
| 57 | Jiangsu | - | - | - | - | - | - |
| 58 | Jiangsu | + | - | + | + | - | + |
| 59 | Jiangsu | - | - | - | - | - | - |
| 60 | Jiangsu | - | - | + | - | - | + |
| 61 | Jiangsu | - | - | - | - | - | - |
| 62 | Jiangsu | - | - | - | - | - | - |
| 63 | Jiangsu | - | - | + | - | - | + |
| 64 | Jiangsu | - | - | - | - | - | - |
| 65 | Jiangsu | - | - | - | - | - | - |
| 66 | Jiangsu | - | - | - | - | - | - |
| 67 | Jiangsu | - | - | - | - | - | - |
| 68 | Jiangsu | - | - | - | - | - | - |
| 69 | Jiangsu | + | - | + | + | - | + |
| 70 | Jiangsu | - | - | - | - | - | - |
| 71 | Jiangsu | + | + | + | + | + | + |
| 72 | Jiangsu | - | - | - | - | - | - |
| 73 | Jiangsu | + | - | + | + | - | + |
| 74 | Jiangsu | + | - | + | + | - | - |
| 75 | Jiangsu | - | - | + | - | - | + |
| 76 | Jiangsu | - | - | - | - | - | - |
| 77 | Jiangsu | + | + | + | + | + | + |
| 78 | Jiangsu | + | + | + | + | + | + |
| 79 | Jiangsu | + | - | + | + | - | + |
| 80 | Jiangsu | - | - | - | - | - | - |
| 81 | Jiangsu | - | - | - | - | - | - |
| 82 | Jiangsu | + | - | + | + | - | + |
| 83 | Jiangsu | + | - | + | + | - | + |

| Samples | Province | qPCR | | | PCR | | |
|---|---|---|---|---|---|---|---|
| | | PEDV | TGEV | PDCoV | PEDV | TGEV | PDCoV |
| 84 | Jiangsu | + | - | - | + | - | - |
| 85 | Jiangsu | + | - | - | + | - | - |
| 86 | Jiangsu | + | - | + | + | - | + |
| 87 | Jiangsu | + | - | + | + | - | + |
| 88 | Jiangsu | + | - | + | + | - | + |
| 89 | Jiangsu | + | - | + | + | - | + |
| 90 | Jiangsu | + | - | - | + | - | - |
| 91 | Jiangsu | + | - | + | + | - | + |
| 92 | Jiangsu | + | - | + | + | - | - |
| 93 | Jiangsu | + | + | + | + | + | + |
| 94 | Jiangsu | + | - | + | + | - | + |
| 95 | Jiangsu | - | - | - | - | - | - |
| 96 | Jiangsu | - | - | - | - | - | - |
| 97 | Jiangsu | + | + | + | + | + | + |
| 98 | Jiangsu | - | - | - | - | - | - |
| 99 | Jiangsu | + | - | + | + | - | + |
| 100 | Jiangsu | + | - | + | + | - | + |
| 101 | Jiangsu | + | - | + | + | - | + |
| 102 | Jiangsu | + | - | - | + | - | - |
| 103 | Jiangsu | + | - | + | + | - | + |
| 104 | Jiangsu | - | - | - | - | - | - |
| 105 | Jiangsu | - | - | + | - | - | + |
| 106 | Jiangsu | - | - | - | - | - | - |
| 107 | Jiangsu | - | - | - | - | - | - |
| 108 | Jiangsu | - | - | - | - | - | - |
| 109 | Jiangsu | - | - | + | - | - | + |
| 110 | Jiangsu | - | - | - | - | - | - |
| 111 | Jiangsu | - | - | - | - | - | - |
| 112 | Jiangsu | - | - | - | - | - | - |
| 113 | Jiangsu | - | - | - | - | - | - |
| 114 | Jiangsu | - | - | - | - | - | - |
| 115 | Jiangsu | - | - | - | - | - | - |
| 116 | Jiangsu | + | - | + | + | - | + |
| 117 | Jiangsu | - | - | - | - | - | - |
| 118 | Jiangsu | - | - | - | - | - | - |
| 119 | Jiangsu | - | - | + | - | - | + |
| 120 | Jiangsu | - | - | - | - | - | - |
| 121 | Jiangsu | - | - | + | - | - | + |
| 122 | Jiangsu | - | - | - | - | - | - |
| 123 | Jiangsu | - | - | - | - | - | - |
| 124 | Jiangsu | + | - | + | + | - | + |
| 125 | Jiangsu | + | - | + | + | - | + |

| Samples | Province | qPCR | | | PCR | | |
|---|---|---|---|---|---|---|---|
| | | PEDV | TGEV | PDCoV | PEDV | TGEV | PDCoV |
| 126 | Jiangsu | - | - | - | - | - | - |
| 127 | Jiangsu | - | - | + | - | - | + |
| 128 | Jiangsu | - | - | + | - | - | + |
| 129 | Jiangsu | - | - | - | - | - | - |
| 130 | Jiangsu | - | - | - | - | - | - |
| 131 | Jiangsu | - | - | - | - | - | - |
| 132 | Jiangsu | + | - | + | + | - | + |
| 133 | Jiangsu | + | - | - | + | - | - |
| 134 | Jiangsu | - | - | - | - | - | - |
| 135 | Jiangsu | + | - | + | + | - | + |
| 136 | Jiangsu | + | - | + | + | - | - |
| 137 | Jiangsu | + | - | + | + | - | + |
| 138 | Jiangsu | - | - | - | - | - | - |
| 139 | Jiangsu | - | - | - | - | - | - |
| 140 | Jiangsu | + | - | + | + | - | + |
| 141 | Jiangsu | - | - | - | - | - | - |
| 142 | Jiangsu | + | - | + | + | - | + |
| 143 | Jiangsu | + | - | + | + | - | + |
| 144 | Jiangsu | - | - | + | - | - | + |
| 145 | Jiangsu | + | + | + | + | + | - |
| 146 | Shandong | + | - | + | + | - | + |
| 147 | Shandong | + | - | + | + | - | + |
| 148 | Shandong | + | - | + | + | - | + |
| 149 | Shandong | + | - | + | - | - | + |
| 150 | Shandong | + | - | + | + | - | + |
| 151 | Shandong | + | - | + | + | - | + |
| 152 | Shandong | + | - | + | + | - | + |
| 153 | Shandong | - | - | - | - | - | - |
| 154 | Shandong | + | - | + | + | - | + |
| 155 | Shandong | + | - | + | + | - | - |
| 156 | Shandong | + | - | + | + | - | + |
| 157 | Shandong | - | - | - | - | - | - |
| 158 | Shandong | + | + | + | + | + | + |
| 159 | Shandong | - | - | - | - | - | - |
| 160 | Shandong | - | - | - | - | - | - |
| 161 | Shandong | - | - | - | - | - | - |
| 162 | Shandong | - | - | - | - | - | - |
| 163 | Shandong | - | - | - | - | - | - |
| 164 | Shandong | - | - | - | - | - | - |
| 165 | Shandong | - | - | - | - | - | - |
| 166 | Shandong | - | - | - | - | - | - |
| 167 | Shandong | - | - | - | - | - | - |

| Samples | Province | qPCR | | | PCR | | |
|---|---|---|---|---|---|---|---|
| | | PEDV | TGEV | PDCoV | PEDV | TGEV | PDCoV |
| 168 | Shandong | - | - | - | - | - | - |
| 169 | Shandong | - | - | - | - | - | - |
| 170 | Shandong | - | - | - | - | - | - |
| 171 | Shandong | - | - | - | - | - | - |
| 172 | Shandong | + | + | + | + | + | + |
| 173 | Shandong | + | + | - | + | + | - |
| 174 | Shandong | - | - | - | - | - | - |
| 175 | Shandong | - | - | - | - | - | - |
| 176 | Shandong | - | - | - | - | - | - |
| 177 | Shandong | - | - | - | - | - | - |
| 178 | Shandong | - | - | - | - | - | - |
| 179 | Shandong | - | - | - | - | - | - |
| 180 | Shandong | - | - | - | - | - | - |
| 181 | Shandong | - | - | - | - | - | - |
| 182 | Shandong | + | - | + | + | - | + |
| 183 | Shandong | + | - | + | + | - | + |
| 184 | Shandong | + | - | - | + | - | - |
| 185 | Shandong | + | - | - | + | - | - |
| 186 | Shandong | + | - | - | + | - | - |
| 187 | Shandong | + | - | - | + | - | - |
| 188 | Shandong | + | - | - | - | - | - |
| 189 | Shandong | + | - | - | + | - | - |
| 190 | Shandong | + | - | - | + | - | - |
| 191 | Shandong | + | - | - | + | - | - |
| 192 | Shandong | + | - | - | + | - | - |
| 193 | Shandong | + | - | - | + | - | - |
| 194 | Shandong | + | - | - | + | - | - |
| 195 | Shandong | + | - | + | + | - | + |
| 196 | Shandong | + | - | + | + | - | - |
| 197 | Shandong | + | - | + | + | - | + |
| 198 | Shandong | + | - | - | + | - | - |
| 199 | Shandong | + | - | - | + | - | - |
| 200 | Shandong | + | - | - | + | - | - |
| 201 | Shandong | + | - | + | + | - | + |
| 202 | Shandong | + | - | - | + | - | - |
| 203 | Shandong | + | - | + | + | - | + |
| 204 | Shandong | - | - | - | - | - | - |
| 205 | Shandong | - | - | - | - | - | - |
| 206 | Shandong | - | - | - | - | - | - |
| 207 | Shandong | + | - | + | + | - | - |
| 208 | Shandong | - | - | - | - | - | - |
| 209 | Shandong | - | - | - | - | - | - |

| Samples | Province | qPCR | | | PCR | | |
|---|---|---|---|---|---|---|---|
| | | PEDV | TGEV | PDCoV | PEDV | TGEV | PDCoV |
| 210 | Guangdong | - | - | - | - | - | - |
| 211 | Guangdong | - | - | - | - | - | - |
| 212 | Guangdong | - | - | - | - | - | - |
| 213 | Guangdong | - | - | - | - | - | - |
| 214 | Guangdong | - | - | - | - | - | - |
| 215 | Guangdong | - | - | - | - | - | - |
| 216 | Guangdong | - | - | - | - | - | - |
| 217 | Guangdong | - | - | - | - | - | - |
| 218 | Guangdong | - | - | - | - | - | - |
| 219 | Guangdong | + | - | - | + | - | - |
| 220 | Guangdong | + | - | - | + | - | - |
| 221 | Guangdong | + | - | - | + | - | - |
| 222 | Guangdong | - | - | - | - | - | - |
| 223 | Guangdong | + | - | - | + | - | - |
| 224 | Guangdong | + | - | - | + | - | - |
| 225 | Guangdong | - | - | - | - | - | - |
| 226 | Guangdong | - | - | - | - | - | - |
| 227 | Guangdong | - | - | - | - | - | - |
| 228 | Guangdong | - | - | - | - | - | - |
| 229 | Guangdong | - | - | - | - | - | - |
| 230 | Guangdong | - | - | - | - | - | - |
| 231 | Guangdong | - | + | - | - | + | - |
| 232 | Guangdong | + | + | + | + | + | + |
| 233 | Guangdong | + | + | + | + | + | + |
| 234 | Guangdong | + | + | + | + | + | + |
| 235 | Guangdong | + | - | + | + | - | + |
| 236 | Guangdong | + | - | + | + | - | + |
| 237 | Guangdong | + | + | + | + | + | + |
| 238 | Guangdong | + | - | + | + | - | + |
| 239 | Guangdong | + | - | + | + | - | + |
| 240 | Guangdong | + | - | + | + | - | + |
| 241 | Guangdong | + | + | + | + | + | + |
| 242 | Guangdong | + | + | + | + | + | - |
| 243 | Guangdong | + | + | + | + | + | + |
| 244 | Guangdong | + | - | + | + | - | + |
| 245 | Guangdong | + | - | + | + | - | + |
| 246 | Guangdong | + | - | + | + | - | + |
| 247 | Guangdong | + | + | + | + | + | + |
| 248 | Guangdong | + | + | + | + | + | + |
| 249 | Guangdong | + | + | + | + | + | + |
| 250 | Guangdong | + | + | + | + | + | + |
| 251 | Guangdong | + | - | + | + | - | + |

| Samples | Province | qPCR | | | PCR | | |
|---|---|---|---|---|---|---|---|
| | | PEDV | TGEV | PDCoV | PEDV | TGEV | PDCoV |
| 252 | Guangdong | - | - | - | - | - | - |
| 253 | Guangdong | + | - | + | + | - | + |
| 254 | Guangdong | + | - | + | + | - | + |
| 255 | Guangdong | + | - | + | + | - | + |
| 256 | Guangdong | + | - | + | + | - | + |
| 257 | Guangdong | + | - | + | + | - | + |
| 258 | Guangdong | + | - | + | + | - | + |
| 259 | Guangdong | + | - | + | + | - | + |
| 260 | Guangdong | + | - | + | + | - | + |
| 261 | Guangdong | + | - | + | + | - | + |
| 262 | Guangdong | + | - | + | + | - | + |
| 263 | Guangdong | + | - | + | + | - | + |
| 264 | Guangdong | + | - | + | + | - | + |
| 265 | Guangdong | + | - | + | + | - | + |
| 266 | Guangdong | + | - | + | + | - | + |
| 267 | Guangdong | + | - | + | + | - | + |
| 268 | Guangdong | + | - | + | + | - | + |
| 269 | Guangdong | - | - | + | - | - | + |
| 270 | Guangdong | - | - | + | - | - | + |
| 271 | Guangdong | - | - | + | - | - | + |
| 272 | Guangdong | - | - | + | - | - | + |
| 273 | Guangdong | - | - | + | - | - | + |
| 274 | Guangdong | - | - | + | - | - | + |
| 275 | Guangdong | - | - | + | - | - | + |
| 276 | Guangdong | - | - | + | - | - | + |
| 277 | Guangdong | - | - | + | - | - | + |
| 278 | Guangdong | - | - | + | - | - | + |
| 279 | Guangdong | - | - | + | - | - | + |
| 280 | Guangdong | - | - | + | - | - | + |
| 281 | Guangdong | - | - | + | - | - | + |
| 282 | Guangdong | - | - | + | - | - | + |
| 283 | Guangdong | - | - | + | - | - | + |
| 284 | Guangdong | - | - | + | - | - | + |
| 285 | Guangdong | - | - | - | - | - | - |
| 286 | Guangdong | - | - | + | - | - | + |
| 287 | Guangdong | - | - | + | - | - | + |
| 288 | Guangdong | - | - | + | - | - | + |
| 289 | Guangdong | + | - | + | + | - | + |
| 290 | Guangdong | + | - | + | + | - | + |
| 291 | Guangdong | - | - | - | - | - | - |
| 292 | Guangdong | - | - | - | - | - | - |
| 293 | Guangdong | - | - | - | - | - | - |

| Samples | Province | qPCR | | | PCR | | |
|---|---|---|---|---|---|---|---|
| | | PEDV | TGEV | PDCoV | PEDV | TGEV | PDCoV |
| 294 | Guangdong | - | - | + | - | - | + |
| 295 | Guangdong | - | - | + | - | - | + |
| 296 | Guangdong | - | - | - | - | - | - |
| 297 | Guangdong | - | - | - | - | - | - |
| 298 | Guangdong | + | - | - | + | - | - |
| 299 | Guangdong | - | - | - | - | - | - |
| 300 | Guangdong | + | - | + | + | - | + |
| 301 | Guangdong | + | - | - | + | - | - |
| 302 | Guangdong | - | - | - | - | - | - |
| 303 | Guangdong | + | - | - | + | - | - |
| 304 | Guangdong | + | - | + | + | - | + |
| 305 | Guangdong | + | - | - | + | - | - |
| 306 | Guangdong | + | - | - | + | - | - |
| 307 | Hubei | - | - | - | - | - | - |
| 308 | Hubei | + | - | - | + | - | - |
| 309 | Hubei | - | - | - | - | - | - |
| 310 | Hubei | - | - | - | - | - | - |
| 311 | Hubei | - | - | - | - | - | - |
| 312 | Hubei | - | + | - | - | + | - |
| 313 | Hubei | + | - | - | + | - | - |
| 314 | Hubei | - | - | - | - | - | - |
| 315 | Hubei | - | - | - | - | - | - |
| 316 | Hubei | - | - | - | - | - | - |
| 317 | Hubei | + | + | + | + | + | + |
| 318 | Hubei | - | - | - | - | - | - |
| 319 | Hubei | - | - | - | - | - | - |
| 320 | Hubei | - | - | - | - | - | - |
| 321 | Hubei | - | - | - | - | - | - |
| 322 | Hubei | + | - | - | + | - | - |
| 323 | Hubei | - | - | - | - | - | - |
| 324 | Hubei | - | - | - | - | - | - |
| 325 | Hubei | - | - | - | - | - | - |
| 326 | Hubei | - | + | - | - | + | - |
| 327 | Hubei | + | - | - | + | - | - |
| 328 | Hubei | - | - | - | - | - | - |
| 329 | Hubei | - | + | + | - | + | + |
| 330 | Hubei | - | - | - | - | - | - |
| 331 | Hubei | - | - | - | - | - | - |
| 332 | Hubei | - | - | - | - | - | - |
| 333 | Hubei | - | + | - | - | + | - |
| 334 | Hubei | - | - | - | - | - | - |
| 335 | Hubei | - | - | - | - | - | - |

| Samples | Province | qPCR | | | PCR | | |
|---|---|---|---|---|---|---|---|
| | | PEDV | TGEV | PDCoV | PEDV | TGEV | PDCoV |
| 336 | Hubei | - | - | - | - | - | - |
| 337 | Hubei | - | - | - | - | - | - |
| 338 | Hubei | - | - | - | - | - | - |
| 339 | Hubei | - | - | - | - | - | - |
| 340 | Hubei | - | - | - | - | - | - |
| 341 | Hubei | - | - | - | - | - | - |
| 342 | Hubei | - | - | - | - | - | - |
| 343 | Hubei | - | - | - | - | - | - |
| 344 | Hubei | - | - | - | - | - | - |
| 345 | Hubei | - | - | + | - | - | + |
| 346 | Hubei | - | - | - | - | - | - |
| 347 | Hubei | - | - | - | - | - | - |
| 348 | Hubei | - | - | - | - | - | - |
| 349 | Hubei | - | - | - | - | - | - |
| 350 | Hubei | - | - | - | - | - | - |
| 351 | Hubei | + | - | - | + | - | - |
| 352 | Hubei | + | - | - | + | - | - |
| 353 | Hubei | + | - | - | + | - | - |
| 354 | Hubei | + | - | - | + | - | - |
| 355 | Hubei | + | - | - | + | - | - |
| 356 | Hubei | + | - | - | + | - | - |
| 357 | Hubei | + | - | - | + | - | - |
| 358 | Hubei | + | - | - | + | - | - |
| 359 | Hubei | + | - | - | + | - | - |
| 360 | Hubei | + | - | - | + | - | - |
| 361 | Hubei | + | - | - | + | - | - |
| 362 | Hubei | + | - | - | + | - | - |
| 363 | Hubei | + | - | - | + | - | - |
| 364 | Hubei | + | - | - | + | - | - |
| 365 | Hubei | + | - | - | + | - | - |
| 366 | Hubei | + | - | - | + | - | - |
| 367 | Hubei | + | - | - | + | - | - |
| 368 | Hubei | + | - | + | + | - | + |
| 369 | Hubei | + | - | - | + | - | - |
| 370 | Hubei | + | - | - | + | - | - |
| 371 | Hubei | + | - | - | + | - | - |
| 372 | Hubei | + | - | - | + | - | - |
| 373 | Hubei | + | - | - | + | - | - |
| 374 | Hubei | + | - | - | + | - | - |
| 375 | Hubei | + | - | - | + | - | - |
| 376 | Hubei | + | - | - | + | - | - |
| 377 | Hubei | + | - | + | + | - | + |

| Samples | Province | qPCR | | | PCR | | |
|---|---|---|---|---|---|---|---|
| | | PEDV | TGEV | PDCoV | PEDV | TGEV | PDCoV |
| 378 | Hubei | + | - | - | + | - | - |
| 379 | Hubei | + | - | - | + | - | - |
| 380 | Hubei | + | - | - | + | - | - |
| 381 | Hubei | + | - | + | + | - | + |
| 382 | Hubei | + | - | + | + | - | + |
| 383 | Hubei | + | - | + | + | - | + |
| 384 | Hubei | + | - | + | + | - | + |
| 385 | Hubei | + | - | + | + | - | + |
| 386 | Hubei | + | - | - | + | - | - |
| 387 | Hubei | + | - | + | + | - | + |
| 388 | Hubei | + | - | + | + | - | + |
| 389 | Hubei | + | - | - | + | - | - |
| 390 | Hubei | + | - | - | + | - | - |
| 391 | Hubei | + | - | - | + | - | - |
| 392 | Hubei | + | - | + | + | - | + |
| 393 | Hubei | + | - | - | + | - | - |
| 394 | Hubei | + | - | + | + | - | + |
| 395 | Hubei | + | - | + | + | - | + |
| 396 | Hubei | + | - | - | + | - | - |
| 397 | Hubei | + | - | + | + | - | + |
| 398 | Hubei | + | - | + | + | - | + |
| 399 | Hunan | + | - | + | + | - | + |
| 400 | Hunan | + | + | + | + | + | + |
| 401 | Hunan | + | + | + | + | + | + |
| 402 | Hunan | + | + | + | + | + | + |
| 403 | Hunan | + | + | + | + | + | + |
| 404 | Hunan | + | + | + | + | + | + |
| 405 | Hunan | - | - | + | - | - | + |
| 406 | Hunan | + | - | + | + | - | + |
| 407 | Hunan | + | - | + | + | - | + |
| 408 | Hunan | + | - | + | + | - | + |
| 409 | Hunan | + | + | + | + | + | + |
| 410 | Hunan | - | - | - | - | - | - |
| 411 | Hunan | - | - | + | - | - | + |
| 412 | Hunan | + | + | + | + | + | + |
| 413 | Hunan | + | + | + | + | + | + |
| 414 | Hunan | + | - | + | + | - | + |
| 415 | Hunan | + | - | + | + | - | + |
| 416 | Hunan | - | - | - | - | - | - |
| 417 | Hunan | + | + | + | + | + | + |
| 418 | Hunan | + | - | + | + | - | + |
| 419 | Hunan | + | - | - | + | - | - |

| Samples | Province | qPCR | | | PCR | | |
|---|---|---|---|---|---|---|---|
| | | PEDV | TGEV | PDCoV | PEDV | TGEV | PDCoV |
| 420 | Hunan | + | - | - | + | - | - |
| 421 | Hunan | + | - | - | + | - | - |
| 422 | Hunan | + | - | - | + | - | - |
| 423 | Hunan | + | + | - | + | + | - |
| 424 | Hunan | + | + | - | + | + | - |
| 425 | Hunan | + | + | - | + | + | - |
| 426 | Hunan | + | - | - | + | - | - |
| 427 | Hunan | + | - | - | + | - | - |
| 428 | Hunan | + | + | - | + | + | - |
| 429 | Hunan | + | + | - | + | + | - |
| 430 | Hunan | + | - | - | + | - | - |
| 431 | Hunan | + | - | - | + | - | - |
| 432 | Hunan | + | + | - | + | + | - |
| 433 | Hunan | + | - | - | + | - | - |
| 434 | Hunan | + | - | - | + | - | - |
| 435 | Hunan | + | + | - | + | + | - |
| 436 | Hunan | + | - | - | + | - | - |
| 437 | Hunan | + | + | - | + | + | - |
| 438 | Hunan | + | + | - | + | + | - |
| 439 | Hunan | + | + | - | + | + | - |
| 440 | Hunan | + | - | - | + | - | - |
| 441 | Hunan | + | + | - | + | + | - |
| 442 | Hunan | + | - | - | + | - | - |
| 443 | Hunan | + | - | - | + | - | - |
| 444 | Hunan | + | - | - | + | - | - |
| 445 | Hunan | + | - | - | + | - | - |
| 446 | Hunan | + | - | - | + | - | - |
| 447 | Hunan | + | - | - | + | - | - |
| 448 | Hunan | + | - | - | + | - | - |
| 449 | Hunan | + | - | - | + | - | - |
| 450 | Hunan | + | - | - | + | - | - |
| 451 | Hunan | + | - | - | + | - | - |
| 452 | Hunan | + | + | - | + | + | - |
| 453 | Hunan | + | + | - | + | + | - |
| 454 | Hunan | + | + | - | + | + | - |
| 455 | Hunan | - | - | + | - | - | + |
| 456 | Hunan | - | - | + | - | - | + |
| 457 | Hunan | - | - | + | - | - | + |
| 458 | Hunan | - | - | + | - | - | + |
| 459 | Hunan | - | - | + | - | - | + |
| 460 | Hunan | - | - | - | - | - | - |
| 461 | Hunan | - | - | + | - | - | + |

| Samples | Province | qPCR | | | PCR | | |
|---|---|---|---|---|---|---|---|
| | | PEDV | TGEV | PDCoV | PEDV | TGEV | PDCoV |
| 462 | Hunan | - | - | + | - | - | + |
